# A prospective randomized study comparing electrochemically deposited hydroxyapatite and plasma-sprayed hydroxyapatite on titanium stems: 55 hips followed for 2 years with RSA and DXA

**DOI:** 10.3109/17453674.2010.548027

**Published:** 2011-02-10

**Authors:** Berte Grimsmo Bøe, Stephan M Röhrl, Tore Heier, Finnur Snorrason, Lars Nordsletten

**Affiliations:** ^1^Department of Orthopaedics, Oslo University Hospital, Ullevål; ^2^Faculty of Medicine, University of Oslo; ^3^Department of Surgery, Diakonhjemmet Hospital, Oslo, Norway

## Abstract

**Background and purpose:**

Plasma-sprayed hydroxyapatite (HA) is a successful coating for fixation of uncemented femoral stems. There may be alternative coatings with advantages in bone remodeling and transport of bone-active substances. We investigated whether an electrochemically deposited hydroxyapatite, Bonemaster (BM), might be a safe alternative in total hip arthroplasty. Our hypothesis was that the new coating would not be inferior to the conventional one.

**Patients and methods:**

50 patients (55 hips) were included. The stem was tapered and porous-coated proximally. On top of the porous coating was either HA or BM. Patients were evaluated postoperatively and after 3, 6, 12, and 24 months to measure fixation by radiostereometric analysis (RSA), bone mineral density by dual-energy X-ray absorptiometry (DXA), and conventional radiography. Clinical evaluation was performed with Harris hip score and Oxford hip score, both preoperatively and after 2 years.

**Results:**

After 2 years, the stems had subsided 0.25 (HA) and 0.28 (BM) mm and there were no statistically significant differences between the groups in any direction, regarding both migration and rotation. The BM group retained significantly more bone than the HA group in Gruen zone 1 during the first 2 years. The Harris and Oxford hip scores were similar in both groups.

**Interpretation:**

Electrochemically deposited hydroxyapatite on an uncemented stem does not appear to be inferior to plasma-sprayed HA regarding clinical and radiological results, bone remodeling, and micromotion after 2 years follow-up.

Aseptic loosening is the most frequent complication of total hip arthroplasty (THA) (Havelin et al. 2000). The long-term survival is thought to depend partly on bone loss or osteolysis in the proximal femur after insertion. Plasma-sprayed hydroxyapatite (HA) coatings appear to give effective fixation in the femur (Hallan et al. 2007). Alternative coatings may, however, influence bone remodeling around the prosthesis and may function as a carrier of bone-active substances.

Bonemaster (BM) (Bonemaster is a registered trademark of Biomet Europe) is an electrochemically deposited hydroxyapatite (EDHA) coating (Rößler et al. 2002). This technique makes it possible to add biological substrates such as antibiotics or adhesion peptides to the coating and still keep the coating very thin compared to plasma-sprayed HA.

The thickness of a hydroxyapatite coating is a compromise between the mechanical properties and dissolution of the coating. A thinner coating minimizes the potential of particle shedding during insertion. Fewer particles in the joint mean less third-body wear and less periprosthetic osteolysis (Peters et al. 1992, Shanbhag et al. 1994, Campbell et al. 1995, McKellop et al. 1995). Thinner coatings also lower the risk of HA delamination and preserve the porosity of the underlying metallic coating of the implant. The irregular implant surface increases the surface area, providing a greater contact and ingrowth area (Sewing et al. 2002). EDHA, as in Bonemaster, forms a needle-like porous structure (Rößler et al. 2001) and enhances early-stage fixation between implant and bone (Ban et al. 1997).

We designed a prospective randomized trial to compare conventional plasma-sprayed HA with electrochemically deposited HA after insertion of an uncemented femoral stem. This is the first clinical trial with the Bonemaster coating. We hypothesized that implants with Bonemaster would achieve the same degree of stability and bone remodeling, and the same clinical outcome as implants with traditionally plasma-sprayed HA.

## Patients and methods

**Figure 1.**
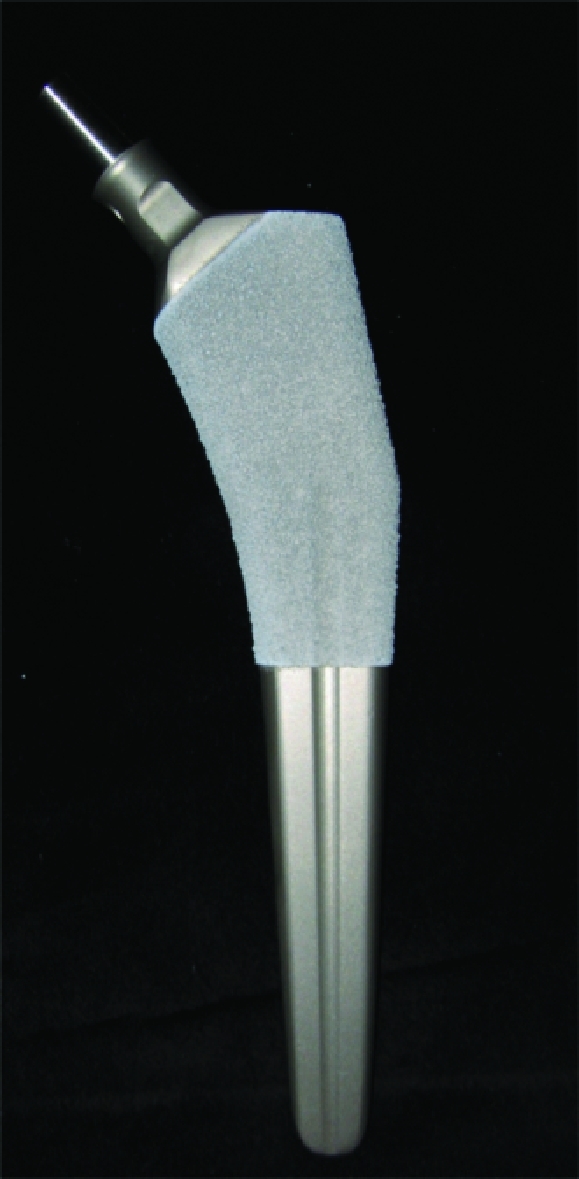
Taperloc stem coated with Bonemaster.

50 patients (31 of whom were women; 55 hips) with noninflammatory end-stage osteoarthritis of the hip participated. Inclusion criteria were health condition expected to allow follow-up for 10 years and anatomy compatible with use of a standard implant. Exclusion criteria were infection, revision arthroplasty, marked bone loss, and severe morbidity. Mean age at the time of operation was 63 (27–81) years. From December 2003 through June 2005, patients underwent THA with the Taperloc uncemented stem (Figure 1), a 28-mm cobalt-chrome modular head and the SHP cemented cup. (The stems were manufactured by Biomet UK Healthcare Ltd.; all other components were from Biomet, Warsaw, IN). Both hips of 5 patients were included. Recruitment was by informed consent and the patients were on our waiting list for THA. The Norwegian Data Inspectorate and the regional ethical committee approved the study and it was carried out in line with the Helsinki declaration.

The patients were randomized (with sealed envelopes) to a stem with either plasma-sprayed HA or Bonemaster. 31 hips were operated with BM-coated stems and 24 with plasma-sprayed HA-coated stems. After a power analysis performed during the study, we ended the inclusion after recruiting 55 hips, leaving 45 envelopes unopened. 1 patient was excluded because of a periprosthetic fracture. 2 patients have subsequently been revised because of loose cups. These 2 patients have been followed with measurements of the stem after their revisions. 1 patient was reoperated after 5 weeks with soft tissue revision and change of femoral head because of infection, but was kept in the study.

4 orthopedic consultants in 2 hospitals operated the patients using the modified Hardinge approach.

The Taperloc stem was manufactured from forged titanium alloy, Ti-6Al-4V. It had a tapered form and was porous-coated proximally. On top of the porous coating, the stem was coated with either plasma-sprayed HA or electrochemically deposited hydroxyapatite (BM). Plasma spraying of HA is a high-temperature process designed to deliver slightly molten Ca(PO_4_)_2_ granules of μm size onto metal surfaces. The process was first described by de Groot et al. (1987). The specifications for the implants in this study were according to the manufacturer: 50-micron thick HA coating (Ca/P ratio: 1.67), a mean surface roughness of 41 microns, a maximum roughness depth of 445 microns, and 62% crystallinity. The electrochemically deposited hydroxyapatite coating was performed in an electrolyte solution near physiological conditions (pH 6.4, 37°C), consisting of 1.67 mM CaCl_2_ and 1 mM NH_4_(H_2_PO_4_) in equal volumes with the implant polarized in cathode galvanostatic mode (–75 A/m2). The layer consisted of 70–72% crystalline HA with the balance being amorphous, and with a thickness of 5 μm and a Ca/P ratio of 2.0. The time taken to apply this form of HA coating is much slower than that of HA applied by plasma spray, and is typically 75 min per implant. Details of preparations, characteristics, and appearances of coatings are as described by Rößler et al. (2002) and Sewing et al. (2004).

On the acetabular side, we used SHP—an all-poly gamma-irradiated (ArCom) cemented cup—inserted with Palacos (Schering-Plough) gentamycin-containing cement.

Bone mineral density (BMD) was measured by experienced technicians using DXA. 3 different DXA machines were used (Prodigy and Expert; both from Lunar, Madison, WI—and Hologic QDR; Hologic Inc., Bedford, MA). The patient was placed supine on the scan table with a foot support to achieve standard rotation of the hip. Orthopedic software (Lunar version 1.2 and Hologic QDR version 12.3) was used to analyze periprosthetic BMD in 7 regions of interest (ROIs). The ROIs were based on the Gruen zones. The patients were measured within a few days postoperatively (mean 5.8 days) and after 3, 6, 12, and 24 months. 30 patients treated at “hospital A” were measured with a Lunar Expert densitometer until January 1, 2005 and later with a Hologic densitometer. 25 patients treated at “hospital B” were measured with a Lunar Prodigy densitometer. We calculated a transformation formula between Lunar Expert and Hologic values based on measurements from 5 of the patients included. These 5 patients were measured twice on both densitometers, and on the same day. Assuming linearity between the 2 machines, the best fit was found using the formula BMD_Lunar_ = 0.789 × (BMD_Hologic_) + 0.2089. Because this transformation of values represents a bias in the patient group from hospital A, we performed statistical analysis on the total number of patients, and on the patients from hospital B separately.

To calculate precision error (the coefficient of variation, CV%) of the 3 densitometers, 130 examinations were repeated on the same day, with repositioning between the scans (Table 1).

**Table 1.** Coefficient of variation (CV) in per cent for the 3 densiometers used in 7 Gruen zones

Zone:	1	2	3	4	5	6	7
Expert	2.0	1.5	2.0	1.4	2.3	2.3	2.8
Hologic	0.5	5.8	1.5	0.6	1.5	1.1	0.8
Prodigy	2.6	2.1	1.7	3.6	2.2	5.1	2.9

### RSA

**Figure 2.**
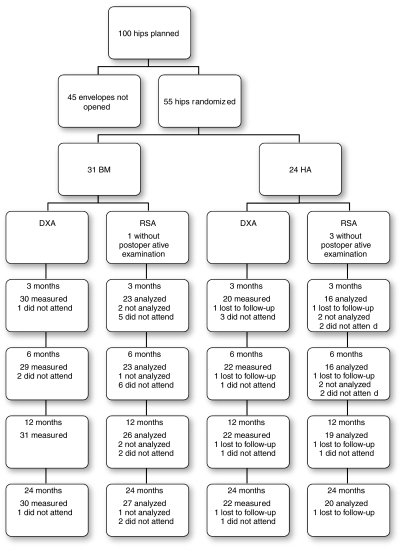
Flow chart of the patients with DXA and RSA measurements. The patient lost to follow-up had an HA-coated stem inserted at hospital A. 17 BM-coated stems were inserted at hospital A and 14 BM-coated stems were inserted at hospital B. 13 HA-coated stems were inserted at hospital A and 11 HA-coated stems were inserted at hospital B. Those patients who were not analyzed by RSA did not meet the criteria of a maximum condition number of 100 or mean error of less than 0.3.

During the operation 7-8 tantalum markers of 1.0 mm were inserted into the proximal part of the femur. The manufacturer had attached 3 tantalum markers to the femoral stem, 1 to the shoulder, 1 to the neck and 1 to the tip. We also computed the position of the femoral head centre. Radiostereometric examinations were done at approximately 7 days, 3 months, 6 months, 1 year, and 2 years after the operation. We evaluated migration of the gravitational centre of the segment which was defined by the stem markers and the centre of the femoral head. Migration was measured along the cardinal axes. Stem rotations were measured as rotations of that segment around the same axes. Analyses were performed using UmRSA (Digital measurement 6.0 RSA Biomedical, Umeå, Sweden). In 4 cases the quality of the postoperative stereoradiographs did not allow a proper evaluation. 1 patient was lost to follow up, 2 did not meet and 1 patient was excluded from the 2 years analysis because of high condition number. 47 patients were to be analyzed at 2 years with mean error below 0.3 and condition number below 100 (Figure 2). 83 examinations were repeated the same day to calculate the precision of our measurements. RSA results are presented as mean values with standard error of mean (SEM).

### Conventional radiography

Anteroposterior and lateral examinations were done preoperatively, postoperatively, and after 3, 6, 12, and 24 months. 2 surgeons evaluated the radiographs. The parameters registered were implant position, radiolucency or lysis, calcar resorption, heterotopic bone formation, trabecular remodeling, cyst formation, pedestal formation, and visible migration. Radiolucent lines were considered to be present if they were > 1.0 mm and occupied more than 50% of the interface in each Gruen zone.

### Clinical evaluation

Harris hip score (Harris 1969) and Oxford hip score (Dawson et al. 1996) were evaluated preoperatively and after 2 years.

### Statistics

The statistical analysis of BMD and RSA results to compare Bonemaster to HA was done using non-inferiority testing comparing areas (SPSS for Mac version PASW 18.0). The BMD results were expressed as percentages (mean) with standard deviation (SD). RSA results were signed values (mean) with SEM. The DXA and RSA results were not normally distributed. The 2 groups were compared with the Mann-Whitney U-test. Changes in DXA, postoperatively to the 2-year follow-up, were analyzed with Wilcoxon signed rank test.

A power analysis was not performed before the study started. Based on an estimated clinically important difference in BMD of 10% (SD 10), stem migration of 0.6 mm (SD 0.6), and stem rotation of 0.7 degrees (SD 0.7) between groups, the sample size calculation indicated 17 patients would be required in each group to achieve 80% power at the 0.05 significance level. Due to the risk of patient dropout, at least 24 patients were included in each group.

## Results

### DXA

After 2 years, there was bone loss compared to the postoperative values in both groups in all regions around the stem (p < 0.05), which was most pronounced in Gruen zones 1 and 7 (Table 2). With non-inferiority testing between the 2 groups, we had to reject the null hypothesis (that Taperloc would function equally well with Bonemaster and HA) for zone 1. Comparison of the areas under the graph showed a significant difference between HA and BM in zone 1 after 2 years (p = 0.01). The bone loss was less in the Bonemaster group. Because of the possible bias with the transformation formula used in hospital A, we also performed the analyses with the results from hospital B alone. We found the same as for the whole group: rejection of the null hypothesis in zone 1 (p = 0.01). For all other Gruen zones, the null hypothesis was retained.

**Table 2.** Periprosthetic changes in bone mineral density around hydroxyapatite- (HA-) and Bonemaster- (BM-) coated Taperloc stems measured by dual-energy X-ray absorbtiometry (DXA). Results are given in percentage (standard deviation) of postoperative values after 3 months, 6 months, 1 year, and 2 years

Gruen zone		n	3 months	n	6 months	n	1 year	n	2 year
1	Bonemaster	30	92 (19)	29	94 (25)	31	90 (26)	30	87 (19)
	HA	20	83 (14)	22	79 (13)	22	76 (15)	22	79 (18)
2	Bonemaster	30	89 (10)	29	92 (8)	31	90 (10)	30	87 (14)
	HA	20	90 (10)	22	90 (8)	22	89 (9)	22	86 (10)
3	Bonemaster	30	93 (7)	29	96 (6)	31	94 (8)	30	90 (12)
	HA	20	94 (5)	22	96 (5)	22	95 (7)	22	92 (9)
4	Bonemaster	30	95 (5)	29	95 (6)	31	94 (5)	30	90 (9)
	HA	20	96 (5)	22	95 (4)	22	94 (6)	22	90 (7)
5	Bonemaster	30	94 (7)	29	97 (7)	31	96 (9)	30	90 (11)
	HA	20	97 (5)	22	97 (6)	22	93 (10)	22	88 (12)
6	Bonemaster	30	93 (9)	29	94 (8)	31	94 (9)	30	89 (11)
	HA	20	94 (7)	22	86 (23)	22	90 (10)	22	88 (9)
7	Bonemaster	30	82 (13)	29	77 (14)	31	73 (15)	30	70 (16)
	HA	20	83 (9)	22	77 (11)	22	73 (12)	22	69 (13)

### RSA

**Figure 3.**
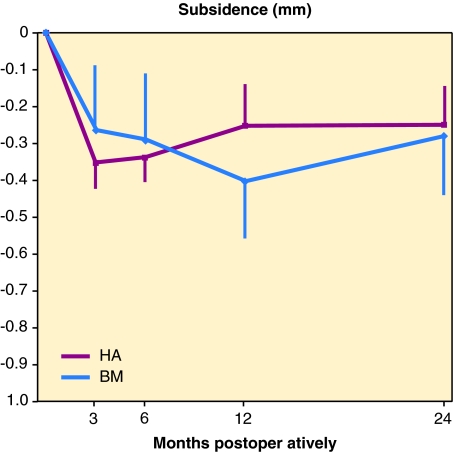
Subsidence (0.25 (HA) and 0.28 (BM) mm in the first 2 years) after implantation of hydroxyapatite- (HA-) and Bonemaster- (BM-) coated Taperloc stems, analyzed by radiostereometric analysis (RSA). Results are given in mm with standard error of the mean after 3 months, 6 months, 1 year, and 2 years.

**Figure 4.**
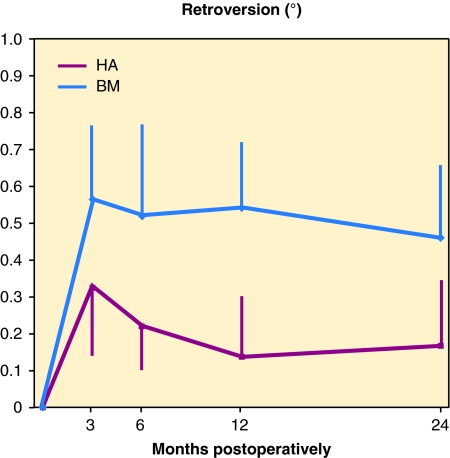
Retroversion of hydroxyapatite- (HA-) and Bonemaster- (BM-) coated Taperloc stems after 2 years, analyzed by radiostereometric analysis. Results are in degrees with standard error of the mean.

The precision of our measurements was 0.11 mm for subsidence and 0.66 degrees for retroversion. The migration pattern for both stems showed that they moved during the first 3 months after surgery and then stabilized (Figures 3 and 4). The mean (SD) subsidence for the center of the stem after 2 years was 0.28 (0.47) mm for BM and 0.25 (0.69) mm for HA (Figure 3). The stems with Bonemaster moved mean 0.46 (0.73) degrees in retroversion compared to 0.17 (0.83) degrees for the HA-coated stems (p = 0.2) (Figure 4). Both groups had retroversion that was lower than the precision in this direction (0.66). With non-inferiority testing, there were no significant differences between groups in any migration or rotation after 2 years.

### Clinical results

Harris hip score increased from 55 (pain score 20) preoperatively to 95 (pain score 41) after 2 years in the Bonemaster group, which was almost similar to the increase from 52 (18) to 89 (38) in the HA group. Oxford hip score improved from 39 preoperatively to 16 after 2 years in the BM group and from 35 to 19 in the HA group. The differences after 2 years were not statistically significant.

There were no radiolucent lines around the stems after 2 years. 1 patient was excluded because of a periprosthetic fracture 6 weeks after the operation. 2 patients in the BM group were revised after 10 and 13 months because of cup loosening. They were both revised because of pain, and we continued to follow the femoral component in the study. Another patient (in the BM group) had a postoperative infection and was revised with soft tissue debridement and irrigation. Treatment of the infection was successful and the patient continued in the study.

RSA and BMD results were analyzed both with and without the reoperated patients included, with no significant changes in the results.

## Discussion

Our study was started as a safety study of a new electrochemically deposited HA coating. The null hypothesis was that Taperloc would function equally well with Bonemaster and HA. Our results show that Bonemaster-coated stems do not function in an inferior way to stems coated with plasma-sprayed HA after 2 years.

The most recent publication for the Taperloc stem without any HA coating (McLaughlin and Lee 2008) describes good long-term results for aseptic loosening with 87% survival after 20 years. The Taperloc is therefore a good stem for safety studies of new technology.

We already knew that HA coating of a porous surface is an excellent fixation method. Both Karrholm et al. (1994) and Soballe et al. (1993) showed that there is less subsidence with HA-coated stems than with porous-coated stems. The question is therefore “do we need Bonemaster?”. The total number of cementless procedures has increased in recent years. A benefit of a coating placed on the stem by galvanic electrolysis is that it can act as a carrier for other substrates. Infections remain a critical issue in total joint arthroplasty. Antibiotics added to the coating would probably lower the infection rate. Alt et al. (2006) demonstrated a lower infection rate in a combined gentamicin-HA group than in an ordinary HA group in a rabbit model.

Osteolysis and bone loss may lead to loosening of the implant or periprosthetic fractures. We suggest that some of the proximal bone loss is due to the surgical trauma from cutting of the neck and preparing the proximal femur to receive the prosthesis. We found less reduction in bone density in the Bonemaster group than in the HA group in Gruen zone 1 during the first 2 years. We found no difference in zones 2, 6, and 7, which are also regions with BM coating. Perhaps the initial difference between the 2 groups was detected in zone 1 because this is an area dominated by trabecular bone. Higher bone density values in the Bonemaster group may indicate a higher degree of bone turnover in trabecular bone in this group. An expected result of DXA measurements after implantation of a hip prosthesis is a marked bone loss initially, and then restoration of bone (Trevisan et al. 1997, Wixson et al. 1997, Karachalios et al. 2004). We have not seen any restoration of bone around the Taperloc stem during the first two years, but bone loss from 7% (zone 3) to 31% (zone 7) is acceptable compared to other stems. It remains to be seen whether the bone loss would continue. For prostheses with satisfactory results, the bone loss is often limited to the proximal zones. In patients with early aseptic loosening, a different pattern of bone remodeling with reduction along the entire stem has been found (Boden et al. 2004). Scott and Jaffe (1996) predicted that higher BMD indicates better ingrowth of bone to the implant. In that case, Bonemaster leads to faster bone ingrowth than HA. This was not reflected by better stability early on, however, as measured by RSA. In an animal study with mechanical pull-out testing, Yang et al. (2008) showed that roughened titanium implants had better initial fixation to bone when they were coated with electrochemically deposited HA than when they only had roughened titanium on the surface.

Another experimental study has shown that plasma-sprayed HA accelerates the early-stage mineralization (< 7 days) of bone more than EDHA coating (Wang et al. 2006). However, EDHA appeared to have resulted in better mechanical integration between the coating and mineralized tissue. Plasma-sprayed HA and EDHA were indistinguishable later (14 days) regarding the mineralized tissue ratio and microstructure they induced in vivo.

Results for the patients measured on the same DXA machine during the entire study were the same as for the whole group. We therefore consider the values to be reliable for comparison, although a change of DXA machine at one institution complicated calculation of bone remodeling.

The amount of acceptable subsidence probably varies between different stem designs and fixation methods. Karrholm et al. (1994) showed that the amount of subsidence after 2 years was the best predictor of later revisions in cemented stems. The cut-off values for the probability of revision to exceed 50% and 95% were 1.2 mm and 2.6 mm of subsidence after 2 years. In a study with HP-Garche uncemented stems, Wykman et al. (1988) reported 0.6–3.9 mm of subsidence in 7 of 8 stems after 2 years. In a later report, the same group reported that 13 of 78 HP-Garche stems (17%) had to be revised in less than four years (Wykman et al. 1991).

Wykman and Lundberg (Wykman and Lundberg 1992) presented an RSA study of 9 patients with porous-coated Taperloc stems. 3 stems had subsided 0.7–0.9 mm after 2 years, and the mean subsidence after 2 years was 0.44 mm. Compared to earlier studies our results thus indicate that concerning subsidence, HA coating of the Taperloc stem is beneficial.

In our trial, the stem subsided in the three first months postoperatively and then stabilized. This migration pattern has also been shown with other HA-coated implants. Thien et al. (2007) published the same pattern of subsidence for 43 ABG stems. For the clinically proven Corail stem, Campbell et al. (2009) documented more subsidence (0.58 mm) than for Taperloc over the first 2 years, using RSA analysis. Corail had the same migration pattern, however, with stability after 6 months. As both Corail and Taperloc have good long-term clinical results (Hallan et al. 2007), it appears that subsidence during the first months and then stabilization within a year is typical for these designs of HA-coated uncemented implants. This might be explained by the ability of hydroxyapatite to close the gap between bone and implant (Overgaard et al. 1997). Long-term follow-up will be necessary to evaluate the effect of electrochemically applied HA on long-term fixation. Further studies are required to investigate whether electrochemically deposited HA combined with antibiotics might lower the infection rate.

In conclusion, the Taperloc stem with Bonemaster does not appear to be inferior to the Taperloc stem with plasma-sprayed HA, concerning clinical and radiological results, bone remodeling, and micromotion—at least up to 2 years.
